# Mutation analysis of congenital cataract in a Chinese family identified a novel missense mutation in the connexin 46 gene (*GJA3*)

**Published:** 2010-04-21

**Authors:** Zhou Zhou, Shanshan Hu, Binbin Wang, Nan Zhou, Shiyi Zhou, Xu Ma, Yanhua Qi

**Affiliations:** 1 Department of Ophthalmology, The Second Affiliated Hospital of Harbin Medical University, Harbin, China; 2 Department of Genetics, National Research Institute for Family Planning, Beijing, China

## Abstract

**Purpose:**

To identify the genetic defects in a three-generation Chinese family with congenital nuclear cataract.

**Methods:**

Four patients and three healthy members from the family underwent complete physical and ophthalmic examinations. Genomic DNA was extracted from peripheral blood leukocytes of the family members as well as from 100 healthy normal controls. Polymerase chain reaction (PCR) amplification and direct sequencing of all coding exons of candidate genes were performed. The functional consequences of the mutation were analyzed with biology softwares.

**Results:**

A novel mutation (c.130G>A) was identified in the connexin 46 gene (*GJA3*), which resulted in the substitution of valine by methionine at the highly conserved codon 44 of connexin 46. This mutation co-segregated among the affected members of the family and was not observed in either unaffected members or the 100 normal controls.

**Conclusions:**

This is a novel missense mutation identified in the first extracellular loop of connexin 46; this expands the mutation spectrum of *GJA3* in association with congenital cataract.

## Introduction

Congenital cataract is a significant cause of poor vision or blindness in children worldwide and is responsible for 10.7%–14.0% of the children who are blind [1]. It is a clinically and genetically heterogeneous lens disorder, with autosomal dominant inheritance being most common. Currently, more than 22 genes have been identified to be associated with various forms of congenital cataract, including ten crystalline genes (*CRYAA* [2], *CRYAB* [3], *CRYBA1/A3* [4], *CRYBA4* [5], *CRYBB1* [6], *CRYBB2* [7], *CRYBB3* [8], *CRYGC* [9], *CRYGD* [10], and *CRYGS* [11]), three transcription factor genes (*HSF4* [12], *PITX3* [13], and *MAF* [14]), two cytoskeletal protein genes (*BFSP1* [15] and *BFSP2* [16]), four membrane transport protein genes (*MIP* [17], *GJA8* [18], *GJA3* [19], and *LIM2* [20]), glucosaminyl (N-acetyl) transferase 2 (*GCNT2*) [21], chromatin-modifying protein-4B (*CHMP4B*) [22], and transmembrane protein 114 (*TMEM114*) [23]. Knowledge of the structure and function of these candidate genes as well as the pathophysiological effect of their disease-associated mutations on their functions will aid in understanding the mechanisms of cataractogenesis.

Here, we report a heterozygous 130G>A transition in the connexin 46 gene (*GJA3*) associated with congenital nuclear cataract in a Chinese family, while it co-segregated completely with the disease phenotype. This is a novel mutation and has not been reported previously with congenital cataract.

## Methods

### Clinical data and sample collection

**Figure 1:**
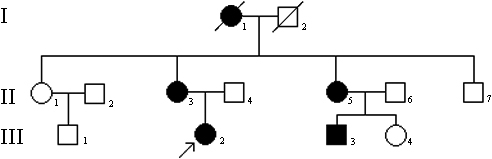
Pedigree of the family. Pedigree of the family with five affected individuals: the proband (III:2), her grandmother (I:1), mother (II:3), aunt (II:5), and male cousin (III:3). Circles represent females, while squares indicate males. Shaded shapes indicate affected individuals. A slash through the symbol indicates the person is deceased. The arrow points to the proband.

**Figure 2:**
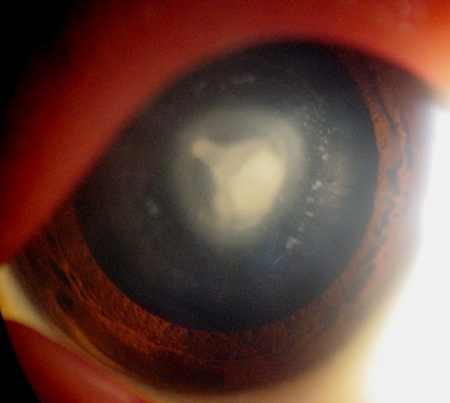
Slit-lamp photograph of the proband. Slit-lamp examination of the proband (III:2) showed a central nuclear cataract involving embryonic and fetal nucleus with punctate cortical opacities.

A three-generation Chinese Han family (Figure 1) with congenital nuclear cataract was recruited from the Second Affiliated Hospital of Harbin Medical University, Harbin, China. Seven members of the pedigree were involved in this study, including four affected individuals (II:3, II:5, III:2, and III:3) and three unaffected ones (II:4, II:6, and III:4). All participants underwent full physical and ophthalmic examinations. Phenotype was documented by slit-lamp photography (Figure 2). One hundred subjects without diagnostic features of congenital cataract were recruited from the Chinese Han population to serve as normal controls. After informed consent, 5 ml venous blood from family members and controls was collected in a BD Vacutainer (BD, San Jose, CA) containing EDTA. Genomic DNA was extracted by QIAamp DNA Blood Mini Kits (QIAGEN Science, Germantown, MD). The research was approved by the Institutional Review Board of Harbin Medical University and followed the clauses of the Declaration of Helsinki.

### Mutation detection

All coding exons and their flanking regions of the known candidate genes associated with autosomal dominant congenital nuclear cataract, such as *CRYAA*, *CRYAB*, *CRYBA1, CRYBB2*, *CRYGC*, *CRYGD*, *CRYGS*, *GJA3*, and *GJA8,* were amplified by PCR with primers listed in Table 1. The PCR products were sequenced from both directions with the ABI3730 Automated Sequencer (PE Biosystems, Foster City, CA). The sequencing results were analyzed using Chromas (version 2.3) and compared with the reference sequences in the NCBI database.

**Table 1:** The primers used for PCR.

**Exon**	**Forward (5′-3′)**	**Reverse (5′-3′)**	**Product length (bp)**
*CRYAA*-1	5′-AGCAGCCTTCTTCATGAGC-3′	5′-CAAGACCAGAGTCCATCG-3′	584
*CRYAA*-2	5′-GGCAGGTGACCGAAGCATC-3′	5′-GAAGGCATGGTGCAGGTG-3′	550
*CRYAA*-3	5′-GCAGCTTCTCTGGCATGG-3′	5′-GGGAAGCAAAGGAAGACAGA-3′	511
*CRYAB*-1	5‘-AACCCCTGACATCACCATTC-3′	5′-AAGGACTCTCCCGTCCTAGC-3′	250
*CRYAB*-2	5′-CCATCCCATTCCCTTACCTT-3′	5′-GCCTCCAAAGCTGATAGCAC-3′	350
*CRYAB*-3	5′-TCTCTCTGCCTCTTTCCTCA-3′	5′-CCTTGGAGCCCTCTAAATCA-3′	400
*CRYBA1*–1	5′-GGCAGAGGGAGAGCAGAGTG-3′	5′-CACTAGGCAGGAGAACTGGG-3′	550
*CRYBA1*–2	5′-AGTGAGCAGCAGAGCCAGAA-3′	5′-GGTCAGTCACTGCCTTATGG-3′	508
*CRYBA1*–3	5′-AAGCACAGAGTCAGACTGAAGT-3′	5′-CCCCTGTCTGAAGGGACCTG-3′	463
*CRYBA1*–4	5′-GTACAGCTCTACTGGGATTG-3′	5′-ACTGATGATAAATAGCATGAACG-3′	355
*CRYBA1*–5	5′-GAATGATAGCCATAGCACTAG-3′	5′-TACCGATACGTATGAAATCTGA-3′	597
*CRYBA1*–6	5′-CATCTCATACCATTGTGTTGAG-3′	5′-CATCTCATACCATTGTGTTGAG-3′	528
*CRYBB2*–1	5′-GTTTGGGGCCAGAGGGGAGTGGT-3′	5′-TGGGCTGGGGAGGGACTTTCAGTA-3′	350
*CRYBB2*–2	5′-CCTTCAGCATCCTTTGGGTTCTCT-3′	5′-GCAGTTCTAAAAGCTTCATCAGTC-3′	330
*CRYBB2*–3	5′-GTAGCCAGGATTCTGCCATAGGAA-3′	5′-GTGCCCTCTGGAGCATTTCATAGT-3′	360
*CRYBB2*–4	5′-GGCCCCCTCACCCATACTCA-3′	5′-CTTCCCTCCTGCCTCAACCTAATC-3′	230
*CRYBB2*–5	5′-CTTACCCTTGGGAAGTGGCAATGG-3′	5′-TCAAAGACCCACAGCAGACAAGTT-3′	600
*CRYGC*-1	5′-TGCATAAAATCCCCTTACCG-3′	5′-CCTCCCTGTAACCCACATTG-3′	514
*CRYGC*-2	5′-TGGTTGGACAAATTCTGGAAG-3′	5′-CCCACCCCATTCACTTCTTA-3′	430
*CRYGD*-1	5′-CAGCAGCCCTCCTGCTAT-3′	5′-GGGTCCTGACTTGAGGATGT-3′	550
*CRYGD*-2	5′-GCTTTTCTTCTCTTTTTATTTCTGG-3′	5′-AAGAAAGACACAAGCAAATCAGT-3′	308
*CRYGS*-2	5′-GAAACCATCAATAGCGTCTAAATG-3′	5′-TGAAAAGCGGGTAGGCTAAA-3′	575
*CRYGS*-3	5′-AATTAAGCCACCCAGCTCCT-3′	5′-GGGAGTACACAGTCCCCAGA-3′	479
*CRYGS*-4	5′-GACCTGCTGGTGATTTCCAT-3′	5′-CACTGTGGCGAGCACTGTAT-3′	974
*GJA3*–1	5′-CGGTGTTCATGAGCATTTTC-3′	5′-CTCTTCAGCTGCTCCTCCTC-3′	450
*GJA3*–2	5′-GAGGAGGAGCAGCTGAAGAG-3′	5′-AGCGGTGTGCGCATAGTAG-3′	450
*GJA3*–3	5′-TCGGGTTCCCACCCTACTAT-3′	5′-TATCTGCTGGTGGGAAGTGC-3′	300
*GJA8*–1	5′-CCGCGTTAGCAAAAACAGAT-3′	5′-CCTCCATGCGGACGTAGT-3′	420
*GJA8*–2	5′-GCAGATCATCTTCGTCTCCA-3′	5′-GGCCACAGACAACATGAACA-3′	330
*GJA8*–3	5′-CCACGGAGAAAACCATCTTC-3′	5′-GAGCGTAGGAAGGCAGTGTC-3′	350
*GJA8*–4	5′-TCGAGGAGAAGATCAGCACA-3′	5′-GGCTGCTGGCTTTGCTTAG-3′	500

Summary of the primers and products length used for the amplification of the all exons of candidate genes related with nuclear cataract.

### Bioinformatics analysis

The wild-type and mutant connexin 46 (Cx46) protein sequences were analyzed with computer assistance for better understanding the effects of the mutation on its biochemical properties. We used PolyPhen (polymorphism phenotyping), which is based on the position-specific independent counts score derived from multiple sequence alignments of observations [24], to predict whether the amino acid substitution affects protein function. An online bio-software program Misc Protein Analysis was used to compute the hydrophilicity of the wild-type and mutant Cx46.

## Results

### Clinical data

There were five affected people in 13 members of this family (Figure 1). The proband (III:2) was a 5-year-old girl whose grandmother (I:1), mother (II:3), aunt (II:5), and male cousin (III:3) also had poor vision in their childhood. Among them, one (I:1) passed away and two (II:3, II:5) had had cataract extractions before examination. The other subjects had had no operations and showed bilateral cataract characterized as a central nuclear opacity involving embryonic and fetal nucleus with punctate cortical opacities (Figure 2). There was no history of other ocular or systemic abnormalities in the family. To date, all of the affected individuals have had cataract surgery.

### Mutation analysis

**Figure 3:**
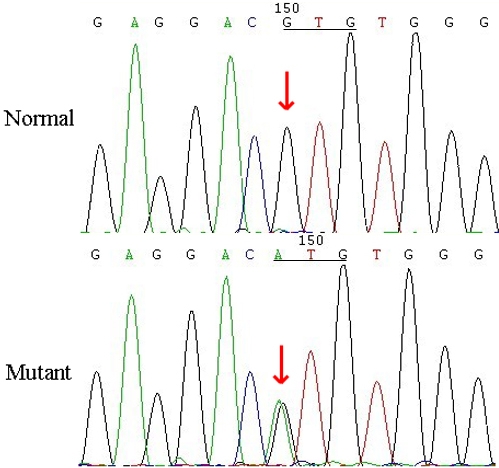
Mutation analysis of the connexin 46 gene (*GJA3*). The sequence chromatogram (forward strand) shows a heterozygous G>A transition that changes valine to methionine at codon 44. The red arrows show the wild-type (normal) and mutant point, respectively.

Direct sequencing of candidate genes revealed a heterozygous G>A transition in *GJA3* at position 130 that led to the replacement of the highly conserved valine with methionine at codon 44 (Figure 3). This mutation was detected in all affected members but was not observed in either the unaffected family members or the normal controls. There was no noticeable nucleotide polymorphism in other candidate genes.

### Bioinformatics analysis

**Figure 4:**
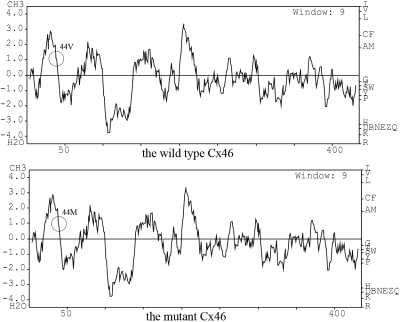
The hydrophilicity of the wild-type and mutant connexin 46 (Cx46). The *x*-axis represents the position of amino acids. The *y*-axis represents the hydrophilicity value in a default window size of nine. The regions of interest are marked by black circles. The decrease in hydrophilicity in the mutant form is evident.

With PolyPhen, substitution in Cx46 at position 44 from V to M scored 1.654 and was confidently predicted to be “possibly damaging.” The obvious decrease in hydrophilicity in the mutant form is shown in Figure 4.

## Discussion

The *GJA3* gene, coding a 435-amino acid protein, was first reported by Willecke et al. [25] in 1990 and is located on chromosome 13q11. Cx46, which is encoded by *GJA3*, is mainly expressed in lens fiber cells. Like others connexins, Cx46 has four transmembrane domains (M1, M2, M3, and M4), two extracellular loops (E1 and E2), an intracellular loop (CL), and intracellular NH_2_ and COOH termini. Cx46 functions as a gap junction that mediates the intercellular transport of small molecules (<1 kDa), including ions, metabolites, and second messengers between elongated fiber cells [26]. Since the lens is an avascular structure and lens fiber cells lose all intracellular organelles during development, the fiber cells are highly dependent on intercellular communication for their survival [27]. The intercellular communication network is formed mainly by the gap junctions. This extensive network is vital since it maintains osmotic and metabolic homeostasis in lens fiber cells and ultimately maintains lens transparency [28].

However, extracellular domains of connexins that contain two extracellular loops (E1 and E2) play a key role in both mediating hemichannel docking [29,30] and regulating voltage gating of the channel [31]. The two extracellular loops are the most conserved domains among connexins and are the sites that provide the strong interaction between the two hemichannels that enable the formation of an intercellular channel with no leakage of current and molecules to the extracellular environment [32]. Furthermore, the first extracellular loop (E1) has been proven to be a major determinant of charge selectivity in Cx46 channels [33].

**Figure 5:**
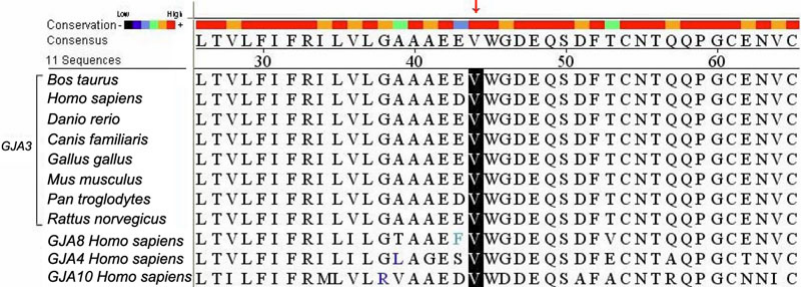
Phylogenetic conservation analysis. Amino acid sequences of connexin 46 (Cx46) from different species and other human connexins were downloaded from the NCBI and automatically aligned by Lasergene MegAlign (DNASTAR, Madison, WI). Multiple alignment indicates that valine at position 44 (black bar highlight) is highly conserved. The red arrow indicates high conservation.

In this study we identified a new mutation (130G>A) in *GJA3*. This variation seems to be disease causative as it segregated with the phenotype and was absent in both unaffected pedigrees and the 100 unrelated controls from a similar ethnic background. This substitution resulted in the replacement of valine to methionine at codon 44 (V44M), localized in the first extracellular loop (E1) of Cx46. A multiple amino acid sequence alignment showed that valine at position 44 is phylogenetically conserved in different species and gap junctions (Figure 5), and Polyphen predicted the mutation to be possibly damaging. These results suggest that valine may be functionally important and the mutation may lead to damaging interference with conformation and function of Cx46. The decline of hydrophilicity in the mutant (Figure 4) might alter the charge on the surface of the extracellular loop, thereby affecting hemichannel docking [34]. The mutation may also affect the charge selectivity in Cx46 channels, disturbing the charge balance inside the lens fiber cells [33]. These changes would disorder intercellular homeostasis in the lens fiber cells and result in lens nucleus opacity.

To date, 15 mutations in *GJA3* have been reported to be associated with congenital cataract in humans (Table 2) [35-45]. Most of these are described as nuclear or zonular pulverulent types and share genotype–phenotype similarities to some extent. In this study the phenotype also shows a conspicuous nuclear cataract but one that is surrounded with punctate opacities. The difference in the cataract phenotypes associated with *GJA3* may be attributed to the action of modifier genes or environmental factors that could affect the expression of *GJA3* and thus resulting cataract types.

**Table 2:** The summary of previous studies of congenital cataract associated with *GJA3*.

**Mutation**	**Amino acid change**	**Location**	**Cataract type**	**Family origin**	**Reference**
c.7G>T	p.D3Y	NH_2_-terminus	Zonular pulverulent	Hispanic Central American	[35]
c.32T>C	p.L11S	NH_2_-terminus	Ant-egg	Danish	[36]
c.82G>A	p.V28M	First transmembrane domain (M1)	Variable	Indian	[37]
c.96C>A	p.F32L	First transmembrane domain (M1)	Nuclear pulverulent	Chinese	[38]
c.98G>T	p.R33L	First transmembrane domain (M1)	Embryonal nuclear granular	Indian	[39]
c.130G>A	p.V44M	First extracellular loop (E1)	Nuclear	Chinese	Present study
c.134G>C	p.W45S	First extracellular loop (E1)	Bilateral nuclear	Chinese	[40]
c.176C>T	p.P59L	First extracellular loop (E1)	Nuclear punctate	American	[41]
c.188A>G	p.N63S	First extracellular loop (E1)	Zonular pulverulent	Caucasian	[18]
c.226C>G	p.R76G	First extracellular loop (E1)	Total	Indian	[37]
c.227G>A	p.R76H	First extracellular loop (E1)	Nuclear pulverulent	Australian	[42]
c.260C>T	p.T87M	Second transmembrane domain (M2)	Pearl box	Indian	[43]
c.560C>T	p.P187L	Second extracellular loop (E2)	Zonular pulverulent	Caucasian	[44]
c.563A>C	p.N188T	Second extracellular loop (E2)	Nuclear pulverulent	Chinese	[45]
c.1137insC	p.S380fs	COOH-terminus	Zonular pulverulent	Caucasian	[18]

Summary of the mutations identified in *GJA3* provide the different congenital cataract phenotypes with different families belonging to different ethnic groups. Most of these mutations are in accord with autosomal dominant, and the cataract phenotypes are nuclear pulverulent types.

In summary, we described a novel missense mutation (V44M) in *GJA3* that causes congenital cataract in a three-generation Chinese family. This study further confirms that Cx46 plays a vital role in the maintenance of human lens transparency and expands the mutation spectrum of *GJA3* in association with congenital cataract.
